# External Validation of Nomograms for Predicting Pelvic Lymph Node Metastases in Patients with Prostate Cancer and the Added Value of the Prostate-specific Membrane Antigen Positron Emission Tomography–based PRIMARY Score

**DOI:** 10.1016/j.euros.2025.10.020

**Published:** 2025-11-11

**Authors:** Muhammet Demirbilek, Göktuğ Kalender, Said Bıyıkoglu, Sertaç Asa, Emre Akkuş, İclal Gürses, Levent Kabasakal, Bülent Önal

**Affiliations:** a Department of Urology, Cerrahpasa Faculty of Medicine, Istanbul University-Cerrahpasa, Istanbul, Turkey; b Department of Nuclear Medicine, Cerrahpasa Faculty of Medicine, Istanbul University-Cerrahpasa, Istanbul, Turkey; c Department of Pathology, Cerrahpasa Faculty of Medicine, Istanbul University-Cerrahpasa, Istanbul, Turkey

**Keywords:** Amsterdam-Brisbane-Sydney nomogram, Pelvic lymph node dissection, PRIMARY score, Prostate cancer

## Abstract

**Background and objective:**

The aim of our study was to predict pathological lymph node involvement (stage pN1) in patients undergoing radical prostatectomy (RP) and extended pelvic lymph node dissection (ePLND) for prostate cancer (PC) and to compare the performance of nomograms used in ePLND decision-making.

**Methods:**

Data for 191 patients with PC who underwent and ePLND between 2018 and 2023 were analyzed retrospectively. Demographics, prostate-specific membrane antigen (PSMA) positron emission tomography (PET)/computed tomography (CT) results, and multiparametric magnetic resonance imaging (mpMRI) findings were assessed in relation to pN1 prediction and nomogram comparison. Statistical analyses included χ^2^ and Mann-Whitney U tests. New models in which PSMA PET/CT parameters (PRIMARY score, mean and maximum intraprostatic standardized uptake values) were incorporated in the Amsterdam-Brisbane-Sydney nomogram were evaluated using the area under the receiver operating characteristic curve (AUC) in a second analysis for a subset of 139 patients. The performance of the new models was analyzed using likelihood ratio tests.

**Key findings and limitations:**

In the primary cohort of 191 patients, 35 (18.3%) had stage pN1 at RP. AUC values were 0.751 (95% confidence interval [CI] 0.676–0.826) for the Briganti 2012 nomogram, 0.722 (95% CI 0.641–0.803) for the Briganti 2017 nomogram, 0.725 (95% CI 0.643–0.807) for the Memorial Sloan Kettering Cancer Center nomogram, and 0.862 (95% CI 0.794–0.929) for the Amsterdam-Brisbane-Sydney nomogram. In the second analysis for the subset of 139 patients, the PRIMARY score was significantly higher in the pN1 group (*p* = 0.011). The AUC for a new model incorporating the PRIMARY score in the Amsterdam-Brisbane-Sydney nomogram was 0.870 (95% CI 0.791–0.949), which surpasses AUC results for the other models. The performance of the newly developed models was significantly better than the original nomogram according to likelihood ratio tests (*p* < 0.001 for all models).

**Conclusions and clinical implications:**

The Amsterdam-Brisbane-Sydney nomogram outperformed other nomograms in predicting pN1 status. The PRIMARY score was significantly higher for a pN1 subgroup versus pN0, which indicates its potential clinical value. These findings suggest that the PRIMARY score and the Amsterdam-Brisbane-Sydney nomogram show promise for pN1 prediction. To the best of our knowledge, this is the first study to investigate the relationship between the PRIMARY score and pN1 status. Further research is needed to clarify the role of the PRIMARY score in clinical decision-making.

**Patient summary:**

We looked at how well different tools predict whether patients with prostate cancer are likely to have metastasis in their pelvic lymph nodes. We found that combining a tool called the Amsterdam-Brisbane-Sydney nomogram with a score for PET (positron emission tomography) scans called the PRIMARY score gave the best prediction performance. This combined tool could help in planning the extent of surgery for patients with intermediate-risk or high-risk prostate cancer if our results are confirmed in other studies.

## Take Home Message

Our study results suggest that the Amsterdam-Brisbane-Sydney nomogram performs better than other models in predicting pathological lymph-node involvement in patients with prostate cancer. The PRIMARY score showed a significant association with lymph node involvement and represents a potential imaging-based marker to support clinical decision-making.

## 1 Introduction

Radical prostatectomy (RP) is one of the most pivotal curative treatment options for localized prostate cancer (PC). Although the impact of pelvic lymph node dissection (PLND) on oncological outcomes remains a subject of debate, its prognostic significance ensures its continued application in patients with intermediate-risk or high-risk PC. A recent study by Touijer et al [1] demonstrated that while ePLND may reduce the risk of nodal metastasis, its benefit regarding biochemical recurrence remains limited. The European Association of Urology (EAU) guidelines recommend that if PLND is to be performed in patients with high-risk PC, an extended PLND (ePLND) template should be used [2]. Similarly, the American Urological Association guidelines recommend ePLND for patients with unfavorable intermediate-risk or high-risk PC, and the use of nomograms in making ePLND decisions [3].

For prediction of pathological lymph node (LN) involvement (pN1 stage), tools such as the Briganti 2012 [4], Briganti 2017 [5], Memorial Sloan Kettering Cancer Center (MSKCC) [6], and Briganti 2019 [7] nomograms are commonly used. While the Briganti 2012, Briganti 2017, and MSKCC nomograms incorporate similar clinicopathological parameters, the Briganti 2019 nomogram includes findings from multiparametric prostate magnetic resonance imaging (mpMRI) and mpMRI-targeted biopsy [7]. However, none of these nomograms include parameters from prostate-specific membrane antigen (PSMA) positron emission tomography (PET)/computed tomography (CT) imaging.

The high performance of PSMA PET in LN staging is well known [8,9]. Building on this high performance, Vis et al [10] developed the Amsterdam-Brisbane-Sydney nomogram, which includes LN involvement status on PSMA PET (molecular imaging: miN0 = no evidence of pelvic LN metastases; miN1 = at least 1 pelvic LN metastasis). The authors reported that this nomogram outperformed the Briganti 2017 and MSKCC nomograms and showed comparable performance to the Briganti 2019 nomogram. Previous studies also reported a correlation between intraprostatic maximum standardized uptake value (SUV_max_) on PSMA PET and pN1 status [11,12]. Furthermore, the recently defined PRIMARY scoring system has highlighted the clinical significance of intraprostatic PSMA uptake patterns in relation to clinically significant PC (csPC) [13]. Given its association with tumor aggressiveness, the PRIMARY score was also considered potentially relevant for predicting pN1 status.

The aim of our study was to examine the performance of nomograms for predicting pN1 stage and investigate the relationships between the PSMA PET–based PRIMARY score and intraprostatic PSMA uptake characteristics and pN1. The ultimate objective was to ensure accurate patient selection for ePLND.

## 2 Patients and methods

### 2.1 Patient population and study protocol

The study included patients diagnosed with PC via standard transrectal ultrasound-guided systematic prostate biopsy (TRUS-Bx) involving 12–18 cores who subsequently underwent either open retropubic RP (ORP) or robot-assisted laparoscopic RP (RARP) and ePLND at our reference center between 2018 and 2023.

External validation of nomograms and investigation of factors predicting pN1 was conducted in a cohort of 191 patients who underwent PSMA PET/CT imaging before surgery. Initially, 240 patients who underwent RARP and ePLND were evaluated. Patients with distant metastases detected on PSMA PET/CT (*n* = 6), those who received preoperative hormone therapy, chemotherapy, or radiotherapy (*n* = 4), and patients with incomplete data (*n* = 39) were excluded. In a subset of 139 patients for whom it was possible to evaluate semiquantitative PSMA PET/CT parameters (PRIMARY score, intraprostatic SUV_max_ and SUV_mean_), the relationship between these PSMA PET/CT parameters and pN1 was explored, and new predictive models were developed.

Ethical approval for the study was obtained from the ethics committee of Istanbul University-Cerrahpasa (reference 06.03.2024-935390), and the study was conducted in accordance with the tenets of the Declaration of Helsinki.

### 2.2 Preoperative nomograms

For the 191 patients in our cohort, pN1 probabilities were calculated using the Briganti 2012 nomogram (preoperative prostate-specific antigen [PSA], cT stage, primary and secondary Gleason scores [GS], and percentage positive cores), the Briganti 2017 nomogram (preoperative PSA, cT stage, biopsy GS, and percentages of positive cores with the highest and lowest grades), the MSKCC nomogram (preoperative PSA, cT stage, primary and secondary GS, and number of negative and positive cores), and the Amsterdam-Brisbane-Sydney nomogram (preoperative PSA, highest grade group from systematic biopsy [no malignancy/no systematic biopsy performed], highest grade group from MRI-targeted biopsy [no malignancy/no systematic biopsy performed], total number of systematic biopsies taken, number of systematic biopsies with grade group ≥2 or higher, radiological stage [rT] on mpMRI, lymph node status on PSMA PET/CT [miN0 or miN1]).

### 2.3 MRI

All patients underwent mpMRI using a 1.5-T or 3.0-T scanner within 2 mo before biopsy. Findings were reported according to Prostate Imaging-Reporting and Data System (PI-RADS) v2.0 or v2.1, depending on the year of application [14,15]. Radiological staging was classified as follows: PI-RADS ≤2 as rT1c, PI-RADS 3–5 without extracapsular extension as rT2, PI-RADS 3–5 with extracapsular extension as rT3a, and PI-RADS 3–5 with seminal vesicle invasion as rT3b. Imaging was reported by different reference centers, including Istanbul University-Cerrahpasa, with no central reading.

### 2.4 PSMA PET imaging

All PSMA PET/CT scans were performed at our center within 1 mo before surgery. The [^68^Ga]PSMA-HBED-CC radioligand was synthesized in the laboratory for PSMA PET/CT imaging. Patients received an average of 233.1 ± 62.9 MBq (6.3 ± 1.7 mCi) of [^68^Ga]PSMA-617 intravenously. PET/CT scans from the vertex to mid-thigh were performed 45–60 min following injection using a Discovery PET-CT 710 device (GE Healthcare, Milwaukee, WI, USA). Low-tube-current CT transmission scans (130 kV peak, 48–76 mA s) were performed without intravenous contrast, with the slice thickness set to 4.0 mm. PET scans were conducted sequentially for 3 min per bed position. CT transmission images were used for attenuation correction.

PET images were reviewed using Advantage Workstation v4.6 (GE Healthcare). The SUV_max_ and SUV_mean_ for LNs in the true pelvis were recorded from the PET images, corresponding to the LNs identified on CT. The region of the prostate with the highest PSMA expression was identified and used to define intraprostatic SUV_max_. Lesions were first located on fusion images, and their corresponding areas on PET scans were delineated using the Auto-Threshold Volume of Interest (VOI) tool (set at 42% of SUV_max_) in Advantage Workstation. Then SUV_max_ and SUV_mean_ values were recorded from within the VOI. All PSMA PET/CT scans were reported by two experienced nuclear medicine specialists blinded to clinicopathological data. PRIMARY scores for intraprostatic uptake were noted, with discrepancies resolved by consulting a third nuclear medicine specialist.

### 2.5 Surgical procedure and histopathological evaluation

All ORP and RARP surgical procedures were performed by experienced urological surgeons at our institution. Bilateral ePLND included the obturator fossa, internal iliac vessels, external iliac vessels, and up to the level of the ureter crossing the common iliac artery. Surgical specimens were evaluated by high-volume uropathologists at our institution in accordance with International Society of Urological Pathology (ISUP) protocols [16]. All palpable LNs and fat blocks were examined for possible microscopic metastases.

### 2.6 Statistical analysis

Results are presented as the frequency and percentage for categorical variables, and as the median and interquartile range (IQR) for continuous variables. The Mann-Whitney U test was used for between-group comparison of continuous variables with a non-normal distribution. A χ^2^ test or Fisher’s exact test was used for comparison of categorical variables, as appropriate.

Receiver operating characteristic (ROC) analysis was conducted for variables clinically anticipated to influence identification of the high-risk group, with pN1 as the reference. The area under the ROC curve (AUC) and the corresponding 95% confidence interval (CI) were calculated. In addition, the incremental discriminative value of adding PSMA PET parameters to the highest-performing nomogram was evaluated by comparing the AUCs of the original and the extended models. The performance of the new models was compared using the likelihood ratio test. Statistical analyses and calculations were conducted using SPSS v28.0 and R Studio v4.3.3. Statistical significance was set at *p* < 0.05.

## 3 Results

### 3.1 Baseline characteristics

A total of 191 patients who underwent RP and ePLND were evaluated to investigate factors predicting pN1 and to examine the performance of various nomograms, of whom 35 (18.3%) were diagnosed with pathologically confirmed LN involvement. The pN1 group had a significantly higher rate miN1 rate on PSMA PET (45.7% vs 4.5%; *p* < 0.001), along with higher percentages of positive cores and ISUP grade group (GG) ≥2 cores, clinical stage, EAU risk group, rT stage, and maximum index lesion diameter on mpMRI (all *p* < 0.05). The median number of LNs removed was 17 (IQR 13–24) in the pN1 group and 15 (IQR 10–20) in the pN0 group (*p* = 0.026; Table 1).

**Table 1:** Demographic data and pathological findings for the primary study cohort (*n* = 191) by pN stage

Parameter	pN0 group (*n* = 156)	pN1 group (*n* = 35)	*p* value^a^
Median age, yr (IQR)	65 (59–70)	65 (59–70)	0.9
Median PSA, median/ml (IQR)	8.6 (5.7–14.8)	10.1 (6.7–18)	0.085
Median PSA density, median/ml/cm^3^ (IQR)	0.22 (0.13–0.38)	0.24 (0.13–0.55)	0.2
Median percentage positive cores at biopsy, % (IQR)	0.44 (0.25–0.64)	0.58 (0.42–0.8)	**0.006**
Median percentage cores with GG ≥2 at biopsy, % (IQR)	0.25 (0.12–0.33)	0.33 (0.25–0.5)	**<0.001**
Biopsy ISUP grade group, *n* (%)			0.3
GG 1	22 (14)	1 (2.9)	
GG 2	55 (35)	12 (34)	
GG 3	46 (30)	10 (29)	
GG 4	24 (15)	9 (26)	
GG 5	9 (6)	3 (9)	
Clinical T stage, *n* (%)			**0.031**
cT1	75 (48)	11 (31)	
cT2	70 (45)	17 (49)	
cT3	11 (7)	7 (20)	
European Association of Urology risk group, *n* (%)			**0.001**
Low risk	11 (7.1)	0 (0)	
Intermediate risk	93 (60)	12 (34)	
High risk	52 (33)	23 (66)	
Radiological T stage on MRI, *n* (%)			**0.009**
rT1	10 (6.4)	1 (2.9)	
rT2	134 (86)	24 (69)	
rT3a	5 (3.2)	2 (5.7)	
rT3b	7 (4.5)	8 (23)	
PI-RADS score, *n* (%)			0.11
PI-RADS 1	3 (1.8)	0 (0)	
PI-RADS 2	7 (4.5)	1 (2.8)	
PI-RADS 3	9 (6.8)	3 (8.6)	
PI-RADS 4	77 (49)	10 (29)	
PI-RADS 5	60 (39)	21 (60)	
Median maximum index lesion diameter, mm (IQR)	13 (10–16)	19 (12–28.5)	**<0.001**
Lymph node status on PSMA PET, *n* (%)			**<0.001**
miN0	149 (96)	19 (54)	
miN1	7 (4)	16 (46)	
Median number of lymph nodes removed (IQR)	15 (10–20)	17 (13–24)	**0.026**

IQR = interquartile range; PSA = prostate-specific antigen; ISUP = International Society of Urological Pathology; GG = grade group; MRI = magnetic resonance imaging; PI-RADS = Prostate Imaging-Reporting and Data System; PSMA = prostate-specific membrane antigen; PET = positron emission tomography.

a The *p* values were calculated using a Mann-Whitney U test for continuous variables and a χ^2^ test for categorical variables. Significant *p* values (<0.05) are denoted in bold font.

### 3.2 Nomogram performance

The performance of the nomograms in terms of AUC was 0.751 (95% CI 0.676–0.826) for Briganti 2012, 0.722 (95% CI 0.641–0.803) for Briganti 2017, 0.725 (95% CI 0.643–0.807) for MSKCC, and 0.862 (95% CI 0.794–0.929) for the Amsterdam-Brisbane-Sydney nomogram. The Amsterdam-Brisbane-Sydney nomogram demonstrated the best diagnostic performance in our cohort (Table 2). Evaluation of the nomogram results (correct decision = avoiding overtreatment; incorrect decision = undertreatment) at different cutoff values revealed that at a cutoff of 7% for the Amsterdam-Brisbane-Sydney nomogram, 96 patients would not have undergone ePLND, which would have missed pN1 status in three of these patients (Table 3).

**Table 2:** Nomogram performance

Nomogram	AUC (95% CI)
Briganti 2012	0.751 (0.676–0.826)
Briganti 2017	0.722 (0.641–0.803)
MSKCC	0.725 (0.643–0.807)
Amsterdam-Brisbane-Sydney	0.862 (0.794–0.929)

AUC = area under the receiver operating characteristic curve (derived using SPSS v28.0); CI = confidence interval; MSKCC = Memorial Sloan Kettering Cancer Center.

**Table 3:** Decision rates by pN status at various cutoff values for the nomograms

Nomogram and cutoff	Pts	Decision by pN status, *n* (%)	
		Overtreatment avoided (correct decision in pN0)	Undertreatment (incorrect decision in pN1)
Briganti 2012			
<3%	21	21 (100)	0 (0)
<5%	64	62 (97)	2 (3)
<7%	82	77 (94)	5 (6)
Briganti 2017			
<3%	43	43 (100)	0 (0)
<5%	74	69 (93)	5 (7)
<7%	80	75 (94)	5 (6)
MSKCC			
<3%	20	20 (100)	0 (0)
<5%	61	59 (97)	2 (3)
<7%	78	71 (91)	7 (9)
Amsterdam-Brisbane-Sydney			
<3%	31	31 (100)	0 (0)
<5%	63	61 (97)	2 (3)
<7%	99	96 (97)	3 (3)

MSKCC = Memorial Sloan Kettering Cancer Center; Pts = patients.

### 3.3 Performance of the Amsterdam-Brisbane-Sydney nomogram on addition of PSMA PET parameters

In a second analysis in the subset of 139 cases with semiquantitative PSMA PET parameters available, we evaluated the performance of new models in which the PSMA PET parameters were added to the best-performing Amsterdam-Brisbane-Sydney nomogram. The pN1 group had significantly higher percentages of positive biopsy cores and GG ≥2 cores, EAU risk group, rT stage, and maximum index lesion diameter (*p* < 0.05). Among the semiquantitative PSMA PET parameters, PRIMARY scores were significantly higher in the pN1 group, with scores of 1–3 not observed in this group (*p* = 0.011). In addition, miN1 positivity and intraprostatic SUV_max_ and SUV_mean_ were significantly higher in the pN1 group (all *p* < 0.05; Table 4).

**Table 4:** Demographic, clinical, and pathological characteristics of the secondary cohort for the new model (*n* = 139) by pN status

Parameter	pN0 group (*n* = 114)	pN1 group (*n* = 25)	*p* value^a^
Median age, yr (IQR)	65 (60–71)	63 (59–69.5)	0.4
Median PSA, ng/ml (IQR)	8.8 (5.7–15)	10.7 (7.2–17.6)	0.09
Median percentage positive cores at biopsy, % (IQR)	0.43 (0.25–0.62)	0.58 (0.44–0.75)	**0.005**
Median percentage cores with GG ≥2 at biopsy, % (IQR)	0.21 (0.11–0.36)	0.33 (0.25–0.54)	**0.001**
ISUP grade group at biopsy, *n* (%)			0.4
GG 1	17 (15)	1 (4)	
GG 2	38 (33)	8 (32)	
GG 3	32 (28)	7 (28)	
GG 4	22 (19)	6 (24)	
GG 5	5 (4.4)	3 (12)	
Clinical T stage, *n* (%)			0.06
cT1	54 (47)	7 (28)	
cT2	52 (46)	13 (52)	
cT3	8 (7)	5 (20)	
European Association of Urology risk group, *n* (%)			**0.025**
Low risk	8 (7)	0 (0)	
Intermediate risk	65 (57)	9 (36)	
High risk	41 (36)	16 (64)	
**Magnetic resonance imaging findings**			
Radiological T stage, *n* (%)			**0.004**
rT1	9 (7.9)	0 (0)	
rT2	97 (85)	17 (68)	
rT3a	3 (2.6)	2 (8)	
rT3b	5 (4.4)	6 (24)	
PI-RADS score, n (%)			0.3
PI-RADS 1	2 (1.8)	0 (0)	
PI-RADS 2	7 (6.1)	0 (0)	
PI-RADS 3	7 (6.2)	2 (8)	
PI-RADS 4	52 (46)	9 (36)	
PI-RADS 5	46 (40)	14 (56)	
Median maximum index lesion diameter, mm (IQR)	13 (10–16)	16 (12–27)	**0.007**
**PSMA positron emission tomography findings**			
Lymph node status, *n* (%)			**<0.001**
miN0	109 (96)	13 (52)	
miN1	5 (4)	12 (48)	
PRIMARY score, *n* (%)			**0.011**
1	6 (5.2)	0 (0)	
2	9 (7.9)	0 (0)	
3	10 (8.8)	0 (0)	
4	53 (47)	11 (44)	
5	36 (32)	14 (56)	
Median intraprostatic SUV_max_ (IQR)	8.6 (5.4–15.6)	13.6 (8–27.2)	**0.004**
Median intraprostatic SUV_mean_ (IQR)	4.5 (2.6–7.7)	7.9 (4.5–15.4)	**0.006**
Median lymph node SUV_max_ (IQR)	5 (3.99–37.9)	19.8 (3–33)	0.8
Median lymph node SUV_mean_ (IQR)	2.76 (2.22–24.98)	12.65 (1.54–20.78)	0.9

IQR = interquartile range; PSA = prostate-specific antigen; ISUP = International Society of Urological Pathology; PI-RADS = Prostate Imaging-Reporting and Data System; PSMA = prostate-specific membrane antigen; SUV = standardized uptake value; SUV_max_ = maximum SUV.

a The *p* values were calculated using the Mann-Whitney U test for continuous variables, and a χ^2^ test for categorical variables. Significant *p* values (<0.05) are denoted in bold font.

Models in which intraprostatic SUV_max_ or SUV_mean_ or the PRIMARY score were added to the Amsterdam-Brisbane-Sydney nomogram (original AUC 0.862, 95% CI 0.794–0.929) were evaluated. The model incorporating the PRIMARY score had the highest AUC of 0.870 (95% CI 0.791–0.949; Table 5). Likelihood ratio tests demonstrated that all of the newly developed models had significantly better performance in comparison to the original nomogram (*p* < 0.001; Supplementary Table 1).

**Table 5:** Performance of the new models

Model	AUC (95% CI)
Amsterdam-Brisbane-Sydney	0.862 (0.794–0.929)
Amsterdam-Brisbane-Sydney + intraprostatic SUV_max_	0.854 (0.768–0.941)
Amsterdam-Brisbane-Sydney + intraprostatic SUV_mean_	0.853 (0.767–0.939)
Amsterdam-Brisbane-Sydney + PET PRIMARY score	0.870 (0.791–0.949)

SUV = standardized uptake value; SUV_max_ = maximum SUV; AUC = area under the receiver operating characteristic curve (derived using SPSS v28.0 and R Studio v4.3.3); CI = confidence interval; PET = positron emission tomography.

## 4 Discussion

In our single-center cohort of patients undergoing RP and ePLND for PC, we evaluated the performance of the Briganti 2012, Briganti 2017, MSKCC, and Amsterdam-Brisbane-Sydney nomograms in predicting pN1, as well as the factors associated with pN1 [[4], [5], [6],^10^]. The Amsterdam-Brisbane-Sydney nomogram outperformed the other three nomograms. The PRIMARY score and intraprostatic SUV_max_ and SUV_mean_ on PSMA PET/CT were significantly higher in the pN1 group. We observed an improvement in the performance of the Amsterdam-Brisbane-Sydney nomogram on addition of the PRIMARY score.

To the best of our knowledge, this is the first study to investigate the relationship between the PRIMARY score and pN1 status. We suggest that preoperative PSMA PET/CT imaging, evaluation of intraprostatic lesions, and PSMA PET/CT–based nomograms should be recommended for LN staging and decision-making regarding ePLND in selected patients for whom RP is planned.

The AUC values for the Briganti 2012, Briganti 2017, and MSKCC nomograms in our cohort were significantly lower than those reported for the original cohorts [[4], [5], [6]]. In the original Amsterdam-Brisbane-Sydney nomogram study, the AUC was 0.81 (95% CI 0.78–0.85) for the study cohort and 0.75 (95% CI 0.69–0.81) for the external validation cohort [10]. In our cohort, the AUC for the Amsterdam-Brisbane-Sydney nomogram was 0.862 (95% CI 0.794–0.929), demonstrating superior performance in comparison to the original study and external validation cohorts. Differences in patient characteristics, including the increasing number of patients offered active surveillance and the use of RP and ePLND in advanced PC stages, may explain the difference in nomogram results [17].

The pN1 rate was 8.3%, 12%, and 5.2% in the original cohorts for the Briganti 2012, Briganti 2017, and MSKCC nomograms, respectively, and 26.4% in the original cohort for the Amsterdam-Brisbane-Sydney nomogram [[4], [5], [6],^10^]. In our cohort, the performance of the first three nomograms was similar and low, with the Amsterdam-Brisbane-Sydney nomogram showing the best performance. While Vis et al [10] expressed concerns regarding the diagnostic performance of the Amsterdam-Brisbane-Sydney nomogram in cohorts with lower pN1 rates, its performance in our cohort was outstanding (pN1 rate 18.3% in our cohort vs 33.7% in the external validation cohort).

The nomograms have been evaluated in various external validation cohorts. Oderda et al [18] reported AUC values of 0.83, 0.80, and 0.80 for the Briganti 2012, Briganti 2017, and MSKCC nomograms, respectively, in a cohort in which 9.6% of patients had pN1 status. Gandaglia et al [7] reported AUC values of 0.82, 0.82, and 0.81, respectively, for these nomograms in a cohort with a pN1 rate of 12.5%. Blas et al [19] reported AUC values for the Briganti 2012, Briganti 2017, and MSKCC nomograms of 0.74, 0.76, and 0.78, respectively, in a patient population with a pN1 rate of 18.4%. In a cohort in which 27.6% of patients had pN1 status, Hueting et al [20] found an AUC value of 0.75 for the Briganti 2012 and MSKCC nomograms. Studies with lower pN1 rates than in our cohort revealed higher AUC values for the Briganti 2012, Briganti 2017, and MSKCC nomograms, while studies with similar or higher pN1 rates showed lower AUC values. These results indicate that the pN1 rate can influence nomogram performance across comparing series. A possible explanation for the better diagnostic performance of the PSMA PET–based nomogram may lie in its impact on surgical decision-making. Positive PSMA PET findings may guide surgeons to perform more extensive PLND in selected cases, thereby increasing the likelihood of detecting LN metastasis. This highlights a potential verification bias, and a real clinical benefit of incorporating PSMA PET imaging in preoperative risk assessment.

Our evaluation of nomograms at different cutoff values revealed the highest benefit in the high-risk group. However, the Amsterdam-Brisbane-Sydney nomogram provided greater value in the low-risk group and would have avoided ePLND in 99 patients (52%) in our cohort at a risk threshold of ≤7%, with only three (1.6%) pN1 cases overlooked, suggesting that the nomogram can improve patient selection and reduce unnecessary ePLND procedures (Table 3). The Amsterdam-Brisbane-Sydney nomogram can also be used in patients undergoing MRI-targeted and/or systematic biopsies. Considering that the nomogram was developed in a contemporary cohort with a relatively high pN1 rate, it is expected to become increasingly important in decision-making on whether to perform ePLND.

It has been shown that intraprostatic SUV_max_ correlates with csPC, GS, and pN1 stage [11,12,[21], [22], [23]]. Using the PRIMARY scoring system, Emmet et al [13] demonstrated that both intraprostatic SUV_max_ and patterns of involvement were correlated with csPC. Furthermore, recent studies have reported that PRIMARY scores are associated with ISUP grades and csPC [24,25]. Considering these findings, we developed new models in which the PRIMARY score, intraprostatic SUV_max_, or SUV_mean_ were added to the Amsterdam-Brisbane-Sydney nomogram for a subset of 139 patients. The model that included the PRIMARY score demonstrated the highest AUC value (0.870, 95% CI 0.791–0.949). Although this value was only marginally higher than that for the original Amsterdam-Brisbane-Sydney nomogram (AUC 0.862, 95% CI 0.794–0.929) and the CIs were largely overlapping, a likelihood ratio test confirmed that the extended model provided a statistically significant improvement in performance. Similarly, the model incorporating intraprostatic SUV_max_ had an AUC of 0.854 (95% CI 0.768–0.941), which is comparable to the AUC for the original model. While our results suggest a potential contribution of intraprostatic PSMA uptake patterns to model performance, the added complexity of these models should be weighed against their limited incremental benefit in this data set. Our findings support the importance of intraprostatic involvement patterns, as suggested by Emmet et al [13], but further validation is warranted.

Owing to its retrospective nature, our study has some limitations. Although ePLND was performed as described, LN areas could not be evaluated separately. Nonetheless, the number of LNs removed was consistent with the literature, with a median of 15 (IQR 10–20) in the pN0 group and 17 (IQR 13–24) in the pN1 group [7,26,27]. mpMRI was reported by different reference centers, and there was no central reading. As all scans were performed using ^68^Ga-PSMA-11 with a specific PET system and imaging protocol, the generalizability of our nomogram to other tracers and systems may be limited. As all patients were diagnosed using TRUS-Bx, MRI-targeted nomograms, such as the Briganti 2019 [7] and Briganti 2023 [28] nomograms, could not be included in the evaluation. Furthermore, the recent study by Gandaglia et al [29] showed that the Briganti 2023 nomogram outperformed the Amsterdam-Brisbane-Sydney nomogram for patients with miN0 status; however, the performance of these two nomograms could not be directly compared.

Despite the study limitations, all surgical procedures were performed by the same surgical team at a single reference center, contributing to standardization of the surgical procedure. In addition, all pathology assessments were undertaken by specialized uropathologists in the uropathology department. The entire adipose tissue from ePLND specimens was sampled to evaluate potential subcentimeter metastases. Furthermore, semi-quantitative PET findings were standardized using the same radiopharmaceuticals and PET equipment at the same center.

## 5 Conclusions

Nomograms developed in cohorts with low pN1 prevalence appear to have limited value in guiding ePLND decisions for patients with high risk. Given the high diagnostic accuracy of PSMA PET for detection of pN1 disease, PSMA PET–based models such as the Amsterdam-Brisbane-Sydney nomogram offer a rational approach for clinical decision-making. In addition, the PRIMARY score was associated with pN1 status and represents a promising tool for pN1 prediction. This study is the first to demonstrate a link between the PRIMARY score and pN1 stage, and the results provide a basis for evaluating the role of the PRIMARY score in nodal staging in future studies.

These data were presented at the 7th National Urological Surgery Congress, October 16–20, 2024, Girne, Northern Cyprus (oral presentation SS-14) and at the 40th annual European Association of Urology congress, March 21–24, 2025, Madrid, Spain (abstract A0457).

  ***Author contributions:*** Bülent Onal had full access to all the data in the study and takes responsibility for the integrity of the data and the accuracy of the data analysis.

  *Study concept and design*: Önal, Demirbilek.

*Acquisition of data*: Demirbilek, Önal.

*Analysis and interpretation of data*: Önal, Demirbilek, Kalender.

*Drafting of the manuscript*: Önal, Kabasakal, Demirbilek.

*Critical revision of the manuscript for important intellectual content*: Önal, Akkuş, Kabasakal.

*Statistical analysis*: Demirbilek, Kalender.

*Obtaining funding*: None.

*Administrative, technical, or material support*: Gürses, Asa.

*Supervision*: Önal.

*Other*: None.

  ***Financial disclosures:*** Bülent Onal certifies that all conflicts of interest, including specific financial interests and relationships and affiliations relevant to the subject matter or materials discussed in the manuscript (eg, employment/affiliation, grants or funding, consultancies, honoraria, stock ownership or options, expert testimony, royalties, or patents filed, received, or pending), are the following: None.

  ***Funding/Support and role of the sponsor:*** None.

  ***Ethics statement:*** The protocol for this retrospective study was reviewed and approved by the ethics committee of Istanbul University-Cerrahpasa (reference 06.03.2024-935390). Written informed consent was obtained from all participants.

  ***Data sharing statement:*** This article and the Supplementary material files include all the data generated or analyzed during this study. Further enquiries can be directed to the corresponding author.
