# Supplementary material for: External Validation of Nomograms for Predicting Pelvic Lymph Node Metastases in Patients with Prostate Cancer and the Added Value of the Prostate-specific Membrane Antigen Positron Emission Tomography–based PRIMARY Score
Source: Eur Urol Open Sci. 2025 Nov 11;82:170–7. doi: 10.1016/j.euros.2025.10.020 (PMC12657811; doi:10.1016/j.euros.2025.10.020)
Supplement: Supplementary Data 1 [file mmc1.docx]

**Supplementary Table 1 – Likelihood Ratio Test Results**

|  | χ^2^ | *p* value |
| --- | --- | --- |
| Amsterdam-Brisbane-Sydney + Intraprostatic SUV_max_ | 96.2 | **<0.001** |
| Amsterdam-Brisbane-Sydney + Intraprostatic SUV_mean_ | 100.6 | **<0.001** |
| Amsterdam-Brisbane-Sydney + PRIMARY score | 86.1 | **<0.001** |
